# The Relationship Between Poor Glycemic Control and Diaphragmatic Thickness in Adults with Type 2 Diabetes Mellitus: A Cross-Sectional Study

**DOI:** 10.3390/life16050775

**Published:** 2026-05-06

**Authors:** Banu Açmaz, Vasfiye Nihan Burcek, Mahmut Burak Lacin, Nazmiye Serap Biçer, Hilal Horozoğlu, Zuhal Ozer Simsek, Ali Koc, Yasin Simsek

**Affiliations:** 1 Department of Internal Medicine, Kayseri City Hospital, Kayseri 38080, Turkey; serapbicr@gmail.com; 2 Department of Radiology, Kayseri City Hospital, Kayseri 38080, Turkey; vasfiyeburcek@gmail.com (V.N.B.); mblacin@hotmail.com (M.B.L.); radalikoc@gmail.com (A.K.); 3 Department of Neurology, Kayseri City Hospital, Kayseri 38080, Turkey; hilal.horozoglu@saglik.gov.tr; 4 Department of Chest Disease, Kayseri City Hospital, Kayseri 38080, Turkey; drzosimsek@gmail.com; 5 Department of Endocrine and Metabolic Diseases, Kayseri City Hospital, Kayseri 38080, Turkey; yasinsimsek79@gmail.com

**Keywords:** diabetes mellitus, type 2, diaphragm, ultrasonography, respiratory muscles, hemoglobin A, glycosylated

## Abstract

Introduction: Diabetes mellitus (DM) is associated with multiple systemic complications, yet its effects on respiratory muscle structure remain insufficiently characterized. This study aimed to evaluate diaphragmatic morphology in patients with type 2 DM and to determine whether glycemic control is associated with diaphragmatic thickness. Methods: A total of 120 participants were enrolled, including 60 patients with type 2 DM and 60 healthy controls. Demographic, biochemical, and diaphragmatic ultrasound parameters were assessed, including right and left diaphragm thickness (DT) during inspiration and expiration, diaphragmatic excursion (DE), and costophrenic angle (CPA). All patients with DM underwent electromyography for evaluation of peripheral neuropathy. Diabetic participants were further stratified according to glycemic control using an HbA1c threshold of 7%. Correlation analyses and multivariable linear regression models adjusted for age and body mass index (BMI) were performed to examine the association between HbA1c and diaphragmatic parameters. Results: Compared with controls, patients with DM were older and had higher fasting glucose levels but lower total cholesterol, whereas BMI and other biochemical parameters were comparable. Peripheral neuropathy was identified in 28.3% of patients with DM, but was not associated with significant differences in DT, DE, or CPA. In the three-group analysis, right and left DT measured during both inspiration and expiration differed significantly among well-controlled DM, poorly controlled DM, and control groups, whereas DE and CPA remained similar. Within the DM cohort, higher HbA1c levels were significantly associated with lower right and left DT values. These inverse associations remained independent after adjustment for age and BMI, while no independent associations were observed between HbA1c and either DE or CPA. Conclusions: Poorer glycemic control was associated with reduced diaphragmatic thickness in patients with T2DM. Ultrasonographic assessment may offer a non-invasive approach for detecting early respiratory muscle involvement, although its clinical and functional relevance should be validated in future prospective studies.

## 1. Introduction

According to the International Diabetes Federation Diabetes Atlas 11th edition, approximately 589 million adults worldwide were living with diabetes in 2024, and this number is projected to rise substantially by 2050 [1]. The adverse effects of chronic hyperglycemia on the cardiovascular and nervous systems are well established and are recognized as major complications of DM [2]. Obesity, hypertension, and dyslipidemia may also contribute to the development and progression of diabetes-related complications [3].

Increasing attention has recently been directed toward the lung as a potential target organ in DM [4]. A large study conducted at the MJ Healthcare Center in Beijing between 2009 and 2019, including 11,107 participants, demonstrated that for every 1 mmol/L increase in fasting plasma glucose, forced vital capacity (FVC) decreased by approximately 25 mL, forced expiratory volume in one second (FEV1) decreased by 13 mL, and %FVC and %FEV1 decreased by 0.71–1.03% and 0.46–0.72%, respectively [5]. Similarly, Zhang et al. reported a significant decline in both FEV1 and FVC among patients with DM, whereas the FEV1/FVC ratio remained unchanged [6].

Ultrasonography has increasingly been used for structural and functional evaluation of the diaphragm. In recent years, it has been applied to assess diaphragmatic performance in various clinical settings, including the management of intensive care patients with alveolar collapse or prolonged mechanical ventilation, as well as the evaluation of respiratory adaptation in preterm newborns. With advances in high-resolution ultrasonography, parameters such as diaphragm thickness and diaphragmatic excursion have become practical tools for assessing diaphragmatic structure and motion in clinical practice [7].

Poorly controlled DM is associated with increased morbidity and mortality because of its broad range of complications, which are generally classified as microvascular (diabetic retinopathy, neuropathy, and nephropathy) and macrovascular (cardiovascular disease, stroke, and peripheral artery disease) [8]. Beyond these well-known complications, sarcopenia has recently emerged as a common comorbidity in patients with uncontrolled DM [9]. Sarcopenia has been reported to reduce both the mass and function of respiratory muscles; however, whether the diaphragm is affected by this process in diabetes remains unclear [10,11]. Ultrasonography is a valuable imaging modality that can be used both for functional evaluation of the diaphragm and for the assessment of muscle mass [12].

This study aimed to investigate the effects of diabetes on the diaphragm muscle and to evaluate diaphragmatic morphology in patients with well-controlled DM, poorly controlled DM, and DM complicated by peripheral neuropathy.

## 2. Materials and Methods

### 2.1. Study Design

This cross-sectional study was approved by the Non-Interventional Clinical Research Ethics Committee of Kayseri City Hospital (Approval No: 536; Date: 2 December 2021). Written informed consent was obtained from all participants before enrollment. The study was conducted in the Department of Internal Medicine at Kayseri City Hospital, a tertiary referral center, between 2 January 2022 and 8 December 2022. All procedures were performed in accordance with the ethical standards of the institutional and national research committees and the principles of the Declaration of Helsinki. All data were anonymized before analysis to ensure participant confidentiality.

### 2.2. Participants

A total of 120 adults were enrolled in the study and divided into 2 groups. The control group consisted of 60 apparently healthy hospital staff volunteers who underwent routine periodic health screening. None of the control participants had a history of diabetes mellitus, chronic respiratory disease, neuromuscular disorder, or other major systemic illness, and no clinical or laboratory evidence of metabolic abnormality was present at enrollment. The control and T2DM groups were balanced with respect to sex distribution. The diabetes group consisted of 60 patients with type 2 diabetes mellitus (T2DM) diagnosed according to the American Diabetes Association (ADA) criteria [13]. Only patients with a diabetes duration of at least 5 years were included.

Participants in both groups were evaluated in the early morning after an overnight fast of at least 8 h. Height and weight were measured, and body mass index (BMI) was calculated as weight in kilograms divided by height in meters squared (kg/m^2^). Venous blood samples were collected to assess glucose, low-density lipoprotein (LDL) cholesterol, high-density lipoprotein (HDL) cholesterol, total cholesterol, blood urea nitrogen (BUN), creatinine, aspartate aminotransferase (AST), alanine aminotransferase (ALT), and glycated hemoglobin (HbA1c).

### 2.3. Glycemic Subgroup Classification and Neuropathy Assessment

All patients with T2DM were receiving antidiabetic treatment. Because treatment regimens were heterogeneous, medication classes were not analyzed separately and were not included as separate variables in the analysis. Glycemic control was assessed using an HbA1c threshold of 7%, in accordance with ADA recommendations [14]. For subgroup analyses, patients with T2DM were classified as well-controlled (HbA1c < 7%) or poorly controlled (HbA1c ≥ 7%). In addition, all patients with T2DM underwent electromyographic evaluation, and those with electrophysiologically confirmed peripheral neuropathy were analyzed as a separate subgroup.

### 2.4. Electromyographic Evaluation of Peripheral Neuropathy

Electromyography (EMG) was performed in all patients with T2DM under standardized conditions in a hospital room maintained at 25 °C [15,16,17]. Participants were advised to continue their usual medications and to avoid bathing or applying lotion on the day before the examination. EMG examinations were performed by an experienced neurologist (H.H.).

Motor nerve conduction studies included routine peroneal and tibial motor conduction assessments with F-wave analysis. For symmetry, the contralateral side was also examined when necessary. Upper extremity and sensory nerve conduction studies were additionally performed according to standard electrophysiological practice [15,16,17]. Needle EMG was performed both at rest and during voluntary contraction, and spontaneous activity and motor unit action potentials were recorded using standard techniques [15,16,17]. Peripheral neuropathy was defined on the basis of electrophysiological abnormalities consistent with diabetic peripheral neuropathy.

### 2.5. Exclusion Criteria

Exclusion criteria included hypertension; hyperthyroidism, hypothyroidism, hyperparathyroidism, or hypoparathyroidism; hypercalcemia or hypocalcemia; systemic lupus erythematosus or other connective tissue diseases; clinically significant liver, kidney, or heart disease; use of sedatives, alcohol, tobacco, narcotic substances, calcium channel blockers, or antihypertensive medications; thoracic or abdominal masses; ascites; COVID-19 infection; acute upper or lower respiratory tract infection; fever; asthma; emphysema; chronic obstructive pulmonary disease; coal miner occupation; history of thoracic surgery; and inability to hold the breath for a few seconds during ultrasonographic examination.

### 2.6. Ultrasound Assessment of the Diaphragm

Diaphragm ultrasonography was performed using an Aplio 500 ultrasound systemequipped with a PVT 382BT convex probe (1.8–5.5 MHz; Toshiba Medical Systems Corporation, Otawara, Tochigi, Japan). Video recordings were obtained during respiration, and all measurements were performed from the recorded images to improve measurement accuracy. Ultrasonographic examinations were performed by a radiologist (A.K.) who was blinded to the clinical status of the participants. Each diaphragmatic ultrasonographic measurement was obtained once during the examination. All measurements were performed under the real-time supervision of an experienced physician to ensure adherence to the standardized ultrasonographic protocol.

**Figure 1:**
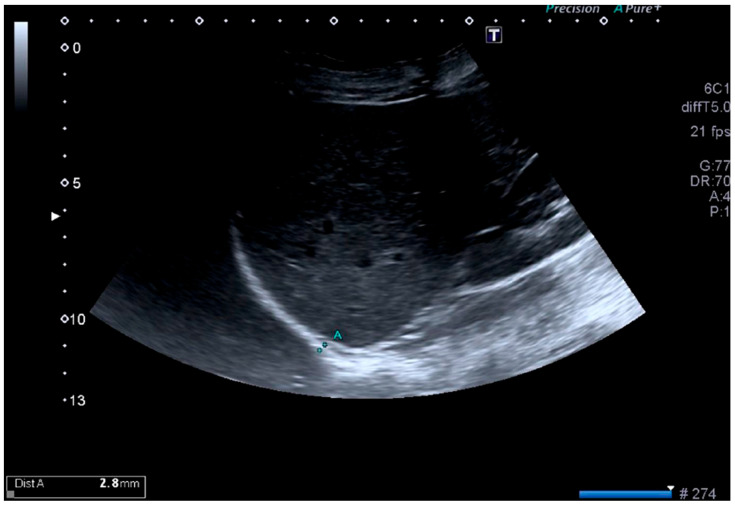
Ultrasonographic measurement of diaphragmatic thickness. Representative B-mode ultrasound image showing diaphragmatic thickness measurement at the zone of apposition. The diaphragm is visualized as a hypoechoic muscle layer between the echogenic pleural and peritoneal lines.

The probe was placed below the costal margin to visualize the zone of apposition of the diaphragm. In this view, the diaphragm was identified as a three-layered structure composed of 2 echogenic lines corresponding to the parietal pleura and peritoneum, with a hypoechoic muscle layer between them. Diaphragm thickness was measured bilaterally during inspiration and expiration, as illustrated in Figure 1. Because the liver may affect visualization and measurement conditions during respiration, the hemidiaphragms were examined separately for thickness measurements, whereas diaphragmatic excursion (DE) was assessed on the left side only to avoid potential hepatic interference. In cases of irregular breathing, coughing, or sneezing, the examination was paused for 1 min before continuing.

### 2.7. Diaphragm Function Parameters

**Figure 2:**
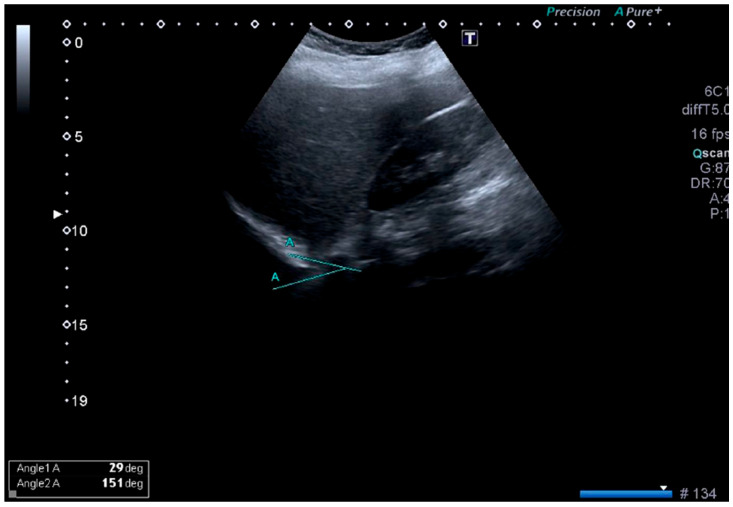
Ultrasonographic measurement of the costophrenic angle (CPA). Representative ultrasound image demonstrating the angle used for CPA assessment.

Diaphragmatic excursion (DE) was defined as the craniocaudal displacement of the diaphragm between the highest and lowest points reached during the respiratory cycle. In addition, changes in the costophrenic angle (CPA) during respiration were evaluated as an indirect marker of diaphragmatic motion (Figure 2) [7,18,19].

### 2.8. Power Analysis

In a pilot analysis, sample size was calculated using diaphragm thickness during inspiration as the primary variable for comparison between the diabetes and control groups. Assuming a power of 90% and a two-sided α level of 0.05, the required sample size was estimated as 27 participants per group. To account for possible dropouts, at least 30 participants per group were targeted [20]. Power analysis was performed using G*Power version 3.1.7.

### 2.9. Statistical Analysis

Statistical analyses were performed using SPSS Statistics version 22.0 (IBM Corp., Armonk, NY, USA). The distribution of continuous variables was assessed using the Shapiro–Wilk test, and homogeneity of variances was evaluated with the Levene test. Continuous variables were expressed as mean ± standard deviation or median (25th–75th percentile), as appropriate. Comparisons between 2 independent groups were performed using the independent-samples *t* test or Mann–Whitney U test, according to data distribution. Comparisons among the 3 study groups were performed using one-way analysis of variance or the Kruskal–Wallis test, as appropriate, followed by pairwise Mann–Whitney U tests for post hoc comparisons when indicated. Correlation analyses were used to evaluate the association between HbA1c and diaphragmatic ultrasound parameters within the T2DM group. In addition, multivariable linear regression analyses adjusted for age and BMI were performed to examine the independent association between HbA1c and diaphragmatic parameters in patients with T2DM. To further address the potential confounding effect of age in the three-group comparison, an additional age-matched sensitivity analysis was performed by selecting equal numbers of participants from each group according to age-group distribution. A two-sided *p* value < 0.05 was considered statistically significant.

## 3. Results

The clinical and biochemical characteristics of the control group (*n* = 60) and patients with diabetes mellitus (DM) (*n* = 60) are presented in Table 1.

Compared with controls, patients with DM were older (49.7 ± 9.5 vs. 43.6 ± 1.7 years, *p* < 0.001) and had higher fasting glucose levels (142 [84–178.5] vs. 87 [84–93.8] mg/dL, *p* < 0.001). Total cholesterol was lower in the DM group than in the control group (179.5 ± 34.2 vs. 194.0 ± 34.2 mg/dL, *p* = 0.030). No significant between-group differences were observed for BUN, creatinine, triglycerides, LDL cholesterol, HDL cholesterol, AST, ALT, or BMI (all *p* > 0.05).

Peripheral neuropathy was identified in 17 of 60 patients with DM (28.3%). Clinical characteristics and diaphragmatic ultrasonography findings according to neuropathy status are shown in Table 2.

Patients with peripheral neuropathy tended to be older than those without neuropathy [57 (45–61) vs. 48 (44–53) years, *p* = 0.057]. HbA1c levels were numerically higher in the neuropathy group [8.4 (7.0–9.1) vs. 7.3 (6.5–8.0), *p* = 0.131], whereas BMI values were similar between groups [31.1 (24.0–38.4) vs. 31.1 (28.4–33.7) kg/m^2^, *p* = 0.902]. Right and left diaphragmatic thickness measurements, diaphragmatic excursion, and costophrenic angle did not differ significantly according to neuropathy status (all *p* > 0.05), although diaphragmatic excursion tended to be lower in patients with neuropathy [15.0 (13.5–17.9) vs. 17.8 (15.0–22.0) mm, *p* = 0.086].

Diaphragmatic ultrasound parameters according to glycemic control status are summarized in Table 3.

When participants were classified into the well-controlled DM (HbA1c < 7%), poorly controlled DM (HbA1c ≥ 7%), and control groups, significant overall group differences were observed for age (*p* = 0.010), right diaphragmatic thickness during inspiration (*p* = 0.003) and expiration (*p* = 0.011), and left diaphragmatic thickness during inspiration (*p* = 0.004) and expiration (*p* = 0.015). BMI, diaphragmatic excursion, and costophrenic angle did not differ significantly across groups (all *p* > 0.05). Post hoc comparisons showed that right and left inspiratory diaphragmatic thickness values were highest in the well-controlled DM group and lowest in the poorly controlled DM group, with the control group showing intermediate values. For right expiratory diaphragmatic thickness, the control group differed from both DM subgroups, whereas the well-controlled and poorly controlled DM groups did not differ from each other. For left expiratory diaphragmatic thickness, the poorly controlled DM group had lower values than both the well-controlled DM and control groups, while the latter two groups were comparable. To further evaluate the potential effect of age imbalance, we performed an additional age-matched sensitivity analysis by selecting 26 participants in each group according to age-group distribution. After matching, the age difference was no longer significant (*p* = 0.986). Importantly, the significant differences in right diaphragmatic thickness during inspiration and expiration and left diaphragmatic thickness during inspiration and expiration remained significant (*p* = 0.009, *p* = 0.011, *p* = 0.016, and *p* = 0.022, respectively), whereas diaphragmatic excursion and costophrenic angle remained non-significant (*p* = 0.481 and *p* = 0.453, respectively).

**Figure 3:**
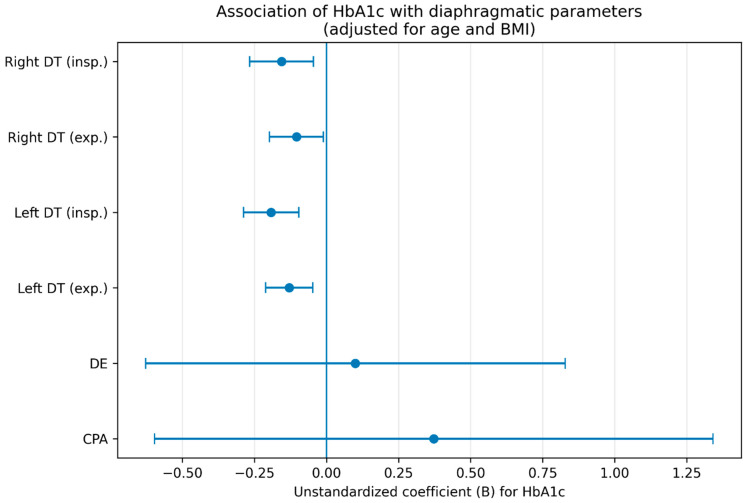
Forest plot of the association between HbA1c and diaphragmatic parameters in patients with diabetes mellitus. Dots represent unstandardized regression coefficients (B), and horizontal lines represent 95% confidence intervals. Estimates were obtained from multivariable linear regression models adjusted for age and BMI. Negative coefficients indicate lower diaphragmatic measurements with increasing HbA1c levels.

Multivariable linear regression analyses adjusted for age and BMI are presented in Table 4 and illustrated in Figure 3.

HbA1c was independently and inversely associated with right diaphragmatic thickness during inspiration (B = −0.156, 95% CI −0.266 to −0.045, *p* = 0.007), right diaphragmatic thickness during expiration (B = −0.104, 95% CI −0.198 to −0.011, *p* = 0.030), left diaphragmatic thickness during inspiration (B = −0.192, 95% CI −0.288 to −0.096, *p* < 0.001), and left diaphragmatic thickness during expiration (B = −0.129, 95% CI −0.211 to −0.047, *p* = 0.003). No independent associations were found between HbA1c and diaphragmatic excursion (*p* = 0.783) or costophrenic angle (*p* = 0.445).

## 4. Discussion

In patients with diabetes mellitus (DM), reductions in FEV1 and FVC are well documented. The impact of diabetes on the respiratory system has gained increasing attention in recent years. Several studies have demonstrated a higher risk of restrictive lung disease in individuals with diabetes, together with an increased prevalence of respiratory conditions such as asthma, COPD, pulmonary fibrosis, and pneumonia [21,22]. These findings suggest that the respiratory system may represent a target of diabetes; however, the mechanisms underlying diabetes-related respiratory involvement remain incompletely understood. Diaphragm thickness has also been investigated in various pulmonary disorders. For example, a magnetic resonance imaging study in patients with COPD reported diaphragmatic dome shortening and flattening, with diaphragm thickness and mobility showing positive correlations with pulmonary function and exercise tolerance, but negative correlations with CAT scores, reflecting poorer quality of life [23]. This suggests that greater diaphragm thickness and mobility may be associated with better clinical outcomes. In our study, we specifically examined the structural and functional effects of diabetes on the diaphragm while excluding patients with known pulmonary disease, thereby allowing a clearer assessment of the potential contribution of glycemic status itself.

The principal finding of the present study is that poorer glycemic control was consistently associated with lower diaphragmatic thickness. When patients were stratified according to glycemic control, significant differences were identified in right and left diaphragmatic thickness measured during both inspiration and expiration, whereas diaphragmatic excursion and costophrenic angle remained comparable across groups. Post hoc analyses further demonstrated a gradient across the study groups: inspiratory diaphragmatic thickness was greatest in the well-controlled DM group, lowest in the poorly controlled DM group, and intermediate in the control group. Considered together with the inverse associations between HbA1c and diaphragmatic thickness, this pattern supports the interpretation that diaphragmatic thinning is linked more closely to the degree of metabolic dysregulation than to the mere presence of diabetes.

This interpretation is strengthened by the correlation and multivariable regression analyses. Within the DM group, HbA1c showed significant inverse correlations with right and left diaphragmatic thickness at both inspiration and expiration. More importantly, these associations remained significant after adjustment for age and BMI. In contrast, HbA1c was not independently associated with diaphragmatic excursion or costophrenic angle. Taken together, these findings suggest that poorer glycemic control is associated with lower diaphragmatic thickness, whereas the relationship with gross diaphragmatic motion appears less evident in this cohort. Although these findings may indicate that structural changes can be detected in association with metabolic dysregulation, the cross-sectional nature of the study does not allow conclusions regarding temporal sequence or causality. Therefore, our results should be interpreted as showing an association between glycemic status and diaphragmatic thickness rather than a direct structural effect of hyperglycemia.

Another important finding of our study is that electrophysiologically defined peripheral neuropathy was not associated with significant differences in diaphragmatic thickness, diaphragmatic excursion, or costophrenic angle. Although patients with neuropathy tended to be older and had numerically lower diaphragmatic excursion values, these differences did not reach statistical significance. Therefore, no significant association between peripheral neuropathy status and diaphragmatic measurements was demonstrated in our cohort. Instead, the overall pattern of our results supports the possibility that diaphragmatic thinning is more closely related to metabolic or myopathic consequences of diabetes. Nevertheless, this finding should be interpreted cautiously, because the neuropathy subgroup was relatively small and subtle neuromuscular effects may have remained undetected.

Survey-based data comparing patients with diabetes to the general population have shown that respiratory symptoms such as dyspnea, chronic cough, and sputum production are more frequent among individuals with diabetes, potentially reflecting an accelerated pulmonary aging process [24]. Although symptomatic patients with known pulmonary disease were excluded from our cohort, the observed association between poorer glycemic control and reduced diaphragm thickness may represent an early marker of structural change in the respiratory musculature. Such thinning may be attributable to diabetes-related alterations in muscle architecture. Insulin resistance and genetic factors are recognized contributors to sarcopenia. Moreover, the accumulation of advanced glycation end-products may adversely affect skeletal muscle quality and contribute to muscle loss in diabetes [25]. Taken together, these mechanisms suggest that the diaphragmatic thinning observed in our study may reflect diabetes-related muscle remodeling and impaired muscle quality. However, this mechanistic interpretation remains inferential and requires confirmation in future studies.

Pulmonary function tests have limited ability to reflect underlying muscle dysfunction. A recent study suggested that diaphragm ultrasonography may be more sensitive than pulmonary function tests in detecting respiratory impairment [26]. In our study, the absence of spirometric data represents a major limitation, as it precluded direct assessment of the functional significance of our findings. Therefore, although reduced diaphragmatic thickness was associated with poorer glycemic control, its direct clinical and functional relevance could not be established in the present study. Additionally, the cross-sectional design limits causal inference, and prospective longitudinal studies are needed to further clarify these associations. Nevertheless, the primary objective of our study was not to evaluate the functional consequences of diaphragmatic changes, but rather to explore the potential structural effects of type 2 diabetes on the diaphragm. In this context, our findings add to the growing body of evidence highlighting the diaphragm as a potential target of diabetes-related muscle alterations.

## 5. Conclusions

Poorer glycemic control was associated with reduced diaphragmatic thickness in patients with T2DM. Ultrasonographic assessment may offer a non-invasive approach for detecting early respiratory muscle involvement, although its clinical and functional relevance should be validated in future prospective studies.

## 6. Limitations

This study has several limitations. First, pulmonary function tests were not performed; therefore, the clinical implications of the observed diaphragmatic changes could not be directly assessed. Nevertheless, patients with known respiratory diseases, such as asthma or COPD, were excluded based on clinical history and physical examination. Second, the diabetic group was significantly older than the control group. Although the regression analyses were adjusted for age and BMI, residual confounding cannot be excluded. In addition, physical activity level and undiagnosed sleep-related breathing disorders were not systematically assessed. Third, because of the cross-sectional design, causal inferences cannot be made. Fourth, the neuropathy subgroup was relatively small, which may have limited our ability to detect subtle differences according to neuropathy status. Fifth, although diaphragmatic thickness was measured bilaterally, diaphragmatic excursion was assessed on the left side only according to the study protocol; therefore, side-specific differences in excursion could not be evaluated. In addition, diaphragmatic ultrasonographic measurements were obtained only once, and formal intra-observer variability was not assessed. Although all measurements were performed under real-time supervision according to a standardized protocol, reproducibility could not be formally evaluated. Furthermore, certain technical factors relevant to reproducibility, such as breathing maneuver standardization and repeated measurement averaging, were not formally analyzed. Finally, antidiabetic treatment regimens were heterogeneous and were not analyzed separately; therefore, possible drug-class-specific effects on diaphragmatic muscle structure may have influenced the observed association between HbA1c and diaphragmatic thickness. In addition, statin use and other lipid-lowering treatments were not systematically recorded; therefore, the lower total cholesterol levels observed in the diabetic group may have reflected treatment effects, and residual confounding related to these medications cannot be excluded. These limitations should be considered in the interpretation of the findings.

## Figures and Tables

**Table 1:** Clinical and biochemical characteristics of the control and diabetes mellitus groups.

Parameter	Control Group (*n* = 60)	DM Group (*n* = 60)	*p* Value
Age (years)	43.6 ± 1.7	49.7 ± 9.5	<0.001
Glucose (mg/dL)	87 (84–93.8)	142 (84–178.5)	<0.001
BUN (mg/dL)	12 (10–14)	13 (11–15)	0.170
Creatinine (mg/dL)	0.8 ± 0.2	0.8 ± 0.2	0.220
TG (mg/dL)	153 (107.5–199)	163 (107.5–199)	0.150
LDL (mg/dL)	108 (98.5–159.5)	107 (74.5–123)	0.090
HDL (mg/dL)	42 (35–48)	43 (32.5–51.5)	0.630
TC (mg/dL)	194 ± 34.2	179.5 ± 34.2	0.030
AST (U/L)	18 (15–22.5)	17 (15–21.5)	0.090
ALT (U/L)	18 (13.5–24)	19 (15–27)	0.480
BMI (kg/m^2^)	32.1 ± 6.7	30.9 ± 5.2	0.270

Values are presented as mean ± standard deviation or median (interquartile range), as appropriate. Abbreviations: DM, diabetes mellitus; BUN, blood urea nitrogen; TG, triglycerides; LDL, low-density lipoprotein; HDL, high-density lipoprotein; TC, total cholesterol; AST, aspartate aminotransferase; ALT, alanine aminotransferase; BMI, body mass index.

**Table 2:** Clinical characteristics and diaphragmatic ultrasonography findings according to peripheral neuropathy status in patients with diabetes mellitus.

Variable	No Neuropathy (*n* = 43)	Neuropathy (*n* = 17)	*p* Value
Age (years)	48 (44–53)	57 (45–61)	0.057
BMI (kg/m^2^)	31.1 (28.4–33.7)	31.1 (24.0–38.4)	0.902
HbA1c (%)	7.3 (6.5–8.0)	8.4 (7.0–9.1)	0.131
Right DT (insp., mm)	3.6 (2.9–4.1)	3.1 (2.9–3.5)	0.233
Right DT (exp., mm)	2.9 (2.4–3.3)	2.5 (1.9–3.1)	0.120
Left DT (insp., mm)	3.7 (2.9–4.0)	3.3 (2.5–3.9)	0.293
Left DT (exp., mm)	2.9 (2.3–3.2)	2.7 (2.3–3.1)	0.439
DE (mm)	17.8 (15.0–22.0)	15.0 (13.5–17.9)	0.086
CPA (°)	34 (32–41)	35 (31–41.5)	0.921

Data are presented as median (25th–75th percentile). Comparisons between patients with and without peripheral neuropathy were performed using the Mann–Whitney U test. Peripheral neuropathy status was determined based on electrophysiological evaluation. DT, diaphragmatic thickness; insp., inspiration; exp., expiration; DE, diaphragmatic excursion; CPA, costophrenic angle.

**Table 3:** Comparison of diaphragmatic ultrasound parameters among well-controlled T2DM, poorly controlled T2DM, and control groups.

Parameter	Well-Controlled DM (HbA1c < 7) (*n* = 30)	Poorly Controlled DM (HbA1c ≥ 7) (*n* = 30)	Control Group (*n* = 60)	*p* Value
Age (years)	50.5 (45.0–57.0) ^a^	48.0 (45.0–56.0) ^ab^	45.0 (35.5–50.0) ^b^	0.010
Sex (male/female), *n*	13/17	17/13	30/30	0.587
BMI (kg/m^2^)	31.3 (28.7–35.0) ^a^	31.1 (27.5–33.8) ^a^	31.5 (29.3–33.3) ^a^	0.771
Right DT (insp., mm)	3.9 (3.4–4.5) ^a^	3.1 (2.8–3.6) ^b^	3.65 (3.1–4.0) ^c^	0.003
Right DT (exp., mm)	3.05 (2.8–3.6) ^a^	2.55 (2.0–2.9) ^a^	2.8 (2.4–3.1) ^b^	0.011
Left DT (insp., mm)	3.8 (3.3–4.1) ^a^	3.1 (2.7–3.8) ^b^	3.4 (3.0–3.8) ^c^	0.004
Left DT (exp., mm)	3.1 (2.7–3.4) ^a^	2.55 (2.2–3.0) ^b^	2.8 (2.3–3.05) ^a^	0.015
DE (mm)	17.2 (14.9–22.0) ^a^	17.0 (14.0–19.3) ^a^	16.05 (15.0–20.0) ^a^	0.691
CPA (°)	33.0 (32.0–41.0) ^a^	35.5 (32.0–41.0) ^a^	36.0 (31.0–43.0) ^a^	0.541

Values are presented as median (25th–75th percentile). Overall *p* values were obtained using the Kruskal–Wallis test. Different superscript letters indicate significant pairwise post hoc differences; groups sharing at least one letter do not differ significantly. Abbreviations: DM, diabetes mellitus; BMI, body mass index; DT, diaphragmatic thickness; insp., inspiration; exp., expiration; DE, diaphragmatic excursion; CPA, costophrenic angle.

**Table 4:** Multivariable linear regression analyses of the association between HbA1c and diaphragmatic parameters in patients with diabetes mellitus.

Dependent Variable	B for HbA1c	95% CI for B	*p* Value	Adjusted R^2^
Right DT (insp., mm)	−0.156	−0.266 to −0.045	0.007	0.127
Right DT (exp., mm)	−0.104	−0.198 to −0.011	0.030	0.055
Left DT (insp., mm)	−0.192	−0.288 to −0.096	<0.001	0.244
Left DT (exp., mm)	−0.129	−0.211 to −0.047	0.003	0.188
DE (mm)	0.100	−0.627 to 0.828	0.783	0.006
CPA (°)	0.372	−0.597 to 1.341	0.445	−0.007

All models were adjusted for age and BMI. Abbreviations: DT, diaphragmatic thickness; insp., inspiration; exp., expiration; DE, diaphragmatic excursion; CPA, costophrenic angle.

## Data Availability

The data are not publicly available due to ethical and privacy restrictions, as they contain clinical information derived from human participants. The anonymized dataset may be made available from the corresponding author upon reasonable request..

## Abbreviations

DM, diabetes mellitus; T2DM, type 2 diabetes mellitus; DE, diaphragmatic excursion; DT, diaphragmatic thickness; CPA, costophrenic angle; FVC, forced vital capacity; FEV1, forced expiratory volume in 1 s; HbA1c, glycated hemoglobin; ADA, American Diabetes Association; BMI, body mass index; LDL, low-density lipoprotein; HDL, high-density lipoprotein; BUN, blood urea nitrogen; AST, aspartate aminotransferase; ALT, alanine aminotransferase; EMG, electromyography; AGEs, advanced glycation end products; TG, triglycerides.
